# Mycetoma Epidemiology and Clinical Findings in Mogadishu, Somalia

**DOI:** 10.1155/jotm/8864108

**Published:** 2025-05-28

**Authors:** Ahmet Doğan, Fadumo Nur Adan, Tigad Abdisad Ali, Ali Kutta Çelik, Ahmed Mohamed Ali

**Affiliations:** ^1^Department of Infectious Diseases and Clinical Microbiology, Abant İzzet Baysal University Faculty of Medicine, Bolu, Turkey; ^2^Department of Infectious Diseases and Clinical Microbiology, Mogadishu-Somalia-Turkey Recep Tayyip Erdoğan Training and Research Hospital, Mogadishu, Somalia; ^3^Department of Infectious Control Nurse, Mogadishu-Somalia-Turkey Recep Tayyip Erdoğan Training and Research Hospital, Mogadishu, Somalia

**Keywords:** actinomycetoma, eumycetoma, Madura foot, Maduromycosis

## Abstract

**Background:** Mycetoma is a public health problem with a high prevalence in Africa.

**Materials and Methods:** The study included 50 cases presenting at a tertiary care research hospital, retrospectively (cases we visited and followed up between November 2022 and March 2023) and prospectively between 1 August and 30 September 2024. Demographic characteristics, clinical features, physical examination findings, and diagnostic methods were reported.

**Results:** Out of 50 patients, 76% were male and 24% were female. The mean age (mean ± SD) of all cases was 35.50 ± 15.14. The most affected occupational group was farmers (44%). All patients presented with complaints of swelling. Symptoms continued for > 1–5 years in about 30 percent of cases. The diagnosis was made by pathological biopsy in 62% of the cases. The lower extremities were most commonly affected (80%), and subcutaneous soft tissue and muscle involvement was also commonly encountered. Bone involvement was higher in eumycetoma cases as compared to actinomycetoma.

**Conclusion:** The frequency of myçetoma cases, which can involve all parts of the lower extremities, was determined, especially in Somali farmers. Difficulties in diagnosis and follow-up were analyzed.

## 1. Introduction

Mycetoma is a chronic, infective, and destructive disease that is particularly common in African populations. Bacteria or fungi are the causative agents. It usually affects the feet with cutaneous or subcutaneous involvement invariably present. On physical examination, multiple discharging sinuses opening onto the skin, tumor-like swellings, and purulent discharge and the commonly encountered findings. Since epidemiological data are limited worldwide, a definite control and prevention program has yet to be developed, with difficulty in the diagnosis being one of the main reasons for this [1–4]. Although it can occur in all age groups, the 20–40 age group is most commonly affected [5]. Males are affected 3 to 4 times more than females [6]. There are studies on the effect of living conditions and other disease risk factors in Africa [7–9]. Precipitation, temperature, soil composition, pH, animal distribution, proximity to water sources, altitude and related topographic variables, and distribution of thorny plant flora are some of the identified factors. Most of the subjects live in rural areas and it has been observed that these patients usually commute near the water bodies. Farming, as an occupation, is a significant risk factor; trauma secondary to stepping barefoot on the contaminated soil acts as a mode of transmission and predisposes to the development of the disease [7–9]. The study aims to analyze the cases admitted to a tertiary care health institution in Mogadishu, Somalia, with a clinical, pathological, radiological, or microbiological diagnosis of mycetoma.

## 2. Materials and Methods

The study was conducted in Mogadishu-Somali-Turkey Recep Tayyip Erdoğan Training and Research Hospital, a tertiary care university hospital. A total of 50 cases were included in the study prospectively and retrospectively (cases we visited and followed up between November 2022 and March 2023). Prospectively, cases admitted to Infectious Diseases, Dermatology, and Orthopedics polyclinics, between 1 August and 30 September 2024 were included. Retrospectively, cases that were previously admitted to our institute, diagnosed, and treated as mycetoma was obtained by reviewing the hospital data processing system. Demographic characteristics, clinical features, physical examination findings, laboratory values, pathological biopsy results and images, and radiological images of all cases were documented. Due to limited resources in the microbiology laboratory, the isolation of pathogens could not be performed in most of the cases. The diagnostic evaluation was primarily based on clinical findings, radiological imaging, and pathology results.

### 2.1. Ethics Committee Approval

An application was made to the Ethics Committee of Mogadishu, Somalia Training and Research Hospital (Approval no: MSTH/18625).

### 2.2. Statistical Analysis

All data were loaded into IBM SPSS Statistics 26.0 (IBM corporation). Frequency and percentage were calculated for categorical variables and mean (standard deviation) and median (interquartile range; IQR) were calculated for continuous variables while the normal distribution evaluation of the data were made with Kolmogorov–Smirnov and Histogram test. The subgroups' significance was determined by the Pearson chi-square test, Fisher's Exact Test. A *p* value of < 0.05 was considered statistically significant.

## 3. Results

Of the 50 cases included in the study, 23 were recorded prospectively, and 27 were recorded retrospectively. Seventy-six percent of cases were male and 24% were female. The mean age of all cases was (mean ± SD) (min-max) 35.50 ± 15.14, (18–81). The most commonly affected occupational group was farmers (44%). No significant comorbidity was detected in 92% of the patients. All patients presented with complaints of swelling. Discharge and pain were second and third most common complaints, respectively. Symptoms continued for > 1–5 years in about 30% of cases. The diagnosis of mycetoma was made by pathological biopsy in 62% of cases. Microbiological diagnosis was possible only in one case. The lower extremities were most commonly affected (80%), and subcutaneous soft tissue and muscle involvement were also commonly seen. Bone involvement was higher in eumycetoma cases compared to actinomycetoma (Table 1).

**Figure 1:**
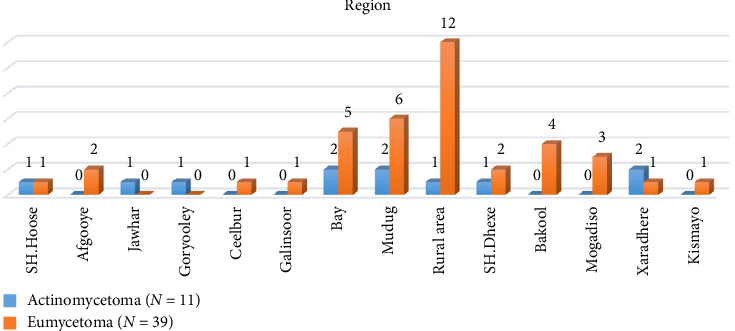
Region-wide distribution of cases in Somalia.

When the regions where the cases lived were analyzed, it was found that they usually lived in remote areas and had difficult access to treatment or health institutions (Figure 1).

**Figure 2:**
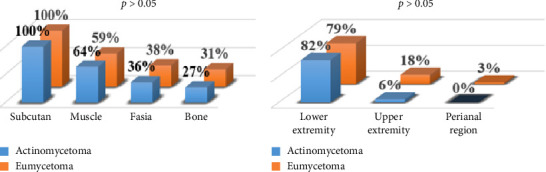
Differential involvement of body parts and tissues as per the causative agent.

When the radiological images of the cases diagnosed by the pathology reports were analyzed, it was observed that muscle involvement was more common in actinomycetoma, while fascia and bone involvement was more common in eumycetoma; and lower extremity involvement was predominant in both groups. The rate of upper extremity involvement was 3 times higher in eumycetoma than in actinomycetoma (Figure 2).

**Figure 3:**
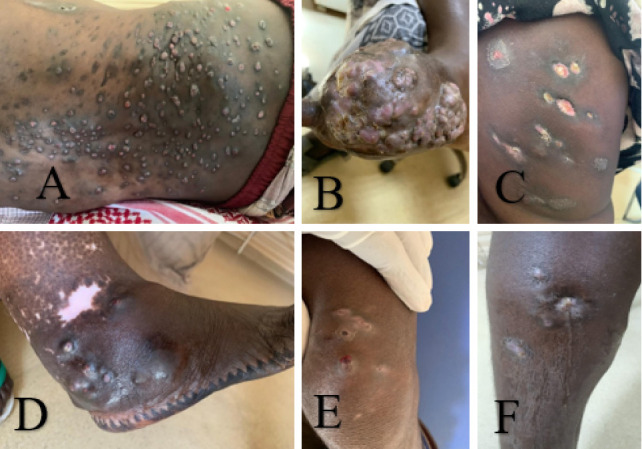
(A, C) Extensive, draining lesions fistulizing to the skin on the dorsal region. (B) Appearance of raised masses on the skin leading to amputation of the toes, more common on the anterior aspect of the right foot. (D) Extensive, draining lesions fistulising to the skin on the lateral aspect of the right foot and (E, F) extensive, draining lesions fistulizing to the skin over the knee joint.

The cases usually presented with multiple discharging sinuses draining\ onto the skin as illustrated by images in Figure 3. The fistulas gradually disappeared in the patients who were under treatment.

**Figure 4:**
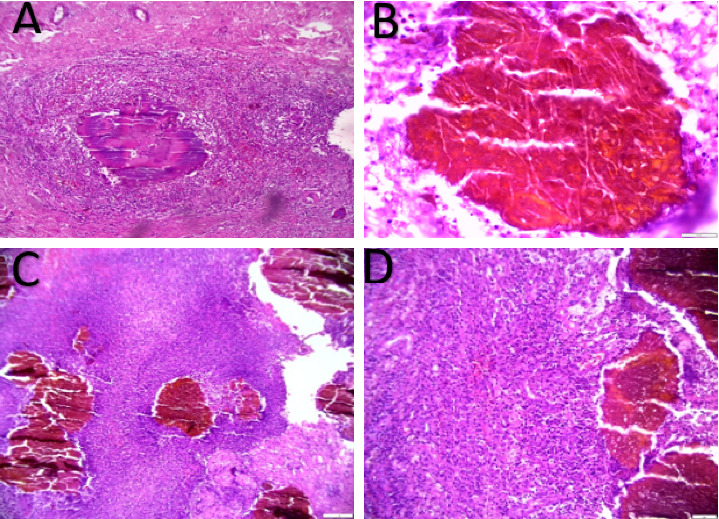
(A) Bacterial actinomycotic colony (grain) outlined by intensely eosinophilic material (Splendore–Hoeppli phenomenon). The surrounding reaction of mixed inflammatory infiltrate comprising lymphocytes, plasma cells, eosinophils, and macrophages with foreign body type giant cells, hematoxylin, and eosin stain, × 40 magnification. (B) Histological sections showing interlacing hyphae and oval club-shaped ends of eumycotic colony 60x. (C, D). Histological sections show a eumycotic colony within a background of mixed inflammatory cells (H and E stain of low power view 10x and 20x).

**Figure 5:**
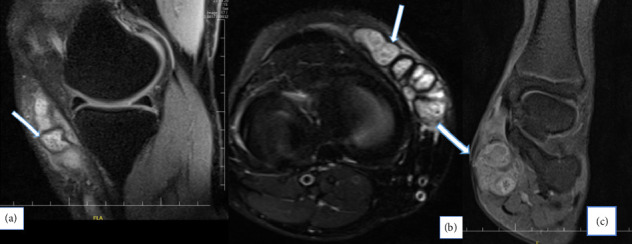
Fluid-sensitive fat-saturated sequences in sagittal (a) and axial (b) planes showing multiple, small, round-to-oval hyperintense lesions with a peripheral hypointense rim. Some of the lesions contain central hypointense dots, resulting in the ‘dot-in-circle' sign (arrows). Coronal contrast-enhanced T1 fat-saturated image (c) showing lobulated peripherally enhancing lesions at the medial plantar aspect of the foot.

Pathological examination and radiological features were also very helpful in determining the diagnosis as illustrated by Figures 4 and 5, respectively.

## 4. Discussion

Apart from a case-based study in Somalia [10], this is the first study on mycetoma conducted on a larger scale. As far as epidemiological factors are concerned, males are affected almost four times more than females. Studies have determined that farmers have a higher risk among the occupational groups, which is understandable as their work involves walking barefoot on soil. When the complaints of the cases coming to the health institutions are examined, there is no complaint of pain in most cases. At the same time, fistulization to the skin and discharge are noticeable [11–13].

Another situation detected in the studies, especially in the African geography, is that eumycetoma cases are seen at a higher rate. People living in rural areas are diagnosed at a very high rate. In regions where soil, thorns, and acacia trees are dense, causative pathogens such as normal flora are a significant risk [14]. Although the lower extremities are primarily affected in most cases, atypical involvement, such as the perianal region, is rarely observed [15]. In our study, lower extremity involvement was generally observed, and perianal participation was observed in one case. Involvement of the upper extremities in eumycetoma and involvement of the back and lumbar region especially in building workers were the prominent findings. The high rate of eumycetoma in the male gender was remarkable. Again, a high rate of swelling was found in eumycetoma cases. The fact that farming and animal breeding are mostly carried out by males may explain the situation. However, it is not possible to explain the distinction between eumycetoma and actinomycetoma based on gender. When the risk factors for eumycetoma and actinomycetoma were evaluated among themselves, no comorbidity was found to make a clinical distinction. No comorbid condition was found in most cases. In the diagnostic methodology, radiological imaging was found to be more useful in eumycetoma. When the duration of symptoms was compared, the duration of symptoms in eumycetoma cases was more than 1 year in about 40% of the cases, while a balanced duration was found in actinomycetoma cases. Similar to literature, a significantly higher rate of mycetoma was found in farmers compared to other occupational groups. Again, like the literature, the number of cases in stockbreeders was significantly higher than in other occupational groups. These occupational groups are usually people living in rural areas. The risk of mycetoma increases due to barefoot walking and minor injuries.

One of the essential factors why the map of mycetoma in the African region is not fully clarified is the difficulties experienced in diagnosis. In addition to the impossibilities of health institutions, isolating the causative pathogen is difficult. Histopathological evaluation, together with clinical evaluation, is the most common diagnostic method. Radiological imaging is essential in diagnosing and determining the degree of organ involvement. It also allows the evaluation of the need for surgery and the response to antimicrobial treatment [16–18]. In our study, the lack of microbiological methods is also noticeable. Histopathological examination was beneficial. Radiological imaging made a vital contribution, especially in evaluating muscle, fascia, and bone involvement. When the locations of the cases seen in the country are analyzed, eumycetoma cases were observed in Bay, Mudung, and especially in rural areas as expected. It is understood that the risk is higher in these regions. To prevent new cases, it is determination obtained from the study that health services should be intensified especially in these regions with information and financial resources.

When the time of presentation of mycetoma cases to health institutions is evaluated, it is seen that there are severe delays. In our study, it was observed that the latest case was admitted after 36 years. It was found that the earliest cases were admitted in 4–5 months, and most were admitted to health institutions between 1 and 5 years. The most important reasons for this are that the cases live in rural areas far away from health institutions and financial inadequacies. However, due to this delay, an increase in deep tissue involvement is seen, especially in eumycetoma cases. Bone involvement has been detected in approximately one-third of the cases, which may mean that cure with antimicrobial treatment alone becomes difficult [19–23].

### 4.1. Study Limitations

Due to the relatively small sample size, the statistically significant difference between the prevalence of eumycetoma and actinomycetoma could not be established. Due to the availability of limited resources at the microbiological lab, the causative pathogen could not be identified/isolated in most cases. Therefore, it was impossible to map the pathogen according to the regions where the cases lived. The study did not include long-term follow-up, treatment response, and the need for surgery.

## 5. Conclusion

In African countries like Somalia, mycetoma remains a significant public health problem. Most of the cases come from rural areas. In almost all cases, fistulization of the skin and discharge are observed. There are severe delays in diagnosis and treatment due to various factors, such as financial constraints. Difficulties in isolation of the pathogen persist. Histopathological examination and clinical evaluation make an essential contribution to the diagnosis. Due to our study's limited number of cases, no significant difference between actinomycetoma and eumycetoma cases could be demonstrated. The risk was found to be relatively high, especially among middle-aged men who were farmers. There is a need to increase the number of microbiological facilities for mapping the isolate type according to the geographical region where the cases live.

## Figures and Tables

**Table 1:** Comparison of cases according to pathology results.

Parameters	Eumycetoma	Actinomycetoma	Total
	*N* = 39, (%)	*N* = 11, (%)	*N* = 50, (%)
*Gender*			
Female	7 (14)	5 (10)	12 (24)
Male	32 (64)	6 (12)	38 (76)

*Profession*			
Housewife	4 (8)	3 (6)	7 (14)
Farmer **(p**=0.049**)**	18 (36)	4 (8)	22 (44)
Stockman	14 (28)	1 (2)	15 (30)
Sheep livestock	1 (2)	3 (6)	4 (8)
Laborer	1 (2)	0 (0)	1 (2)
Driver	1 (2)	0 (0)	1 (2)

*Comorbidity*			
None	35 (70)	11 (22)	46 (92)
DM	1 (2)	0 (0)	1 (2)
HT	1 (2)	0 (0)	1 (2)
DM + HT	2 (4)	0 (0)	2 (4)

*Complaint*			
Swelling	33 (66)	7 (14)	40 (80)
Discharge	13 (26)	4 (8)	17 (34)
Pain	6 (12)	0 (0)	6 (12)

*Duration of symptoms*			
0-1 year	6 (12)	3 (6)	9 (18)
1–5 years	12 (24)	2 (4)	14 (28)
5–10 years	9 (18)	3 (6)	12 (24)
> 10 years	3 (6)	3 (6)	6 (12)

*Diagnostic method*			
Pathology	22 (44)	9 (18)	31 (62)
Radiology	12 (24)	1 (2)	13 (22)
Only clinic	5 (10)	0 (0)	5 (10)
Pathology + microbiology	0 (0)	1 (2)	1 (2)

*Wound culture*			
No	35 (70)	10 (20)	45 (90)
MSSA	2 (4)	0 (0)	2 (4)
MRSA	1 (2)	0 (0)	1 (2)
*Escherichia coli*	0 (0)	1 (2)	1 (2)
*E. coli* + *S. aureus*	1 (2)	0 (0)	1 (2)

*Note:* Percentage calculations were based on total cases (*N* = 50). When eumycetoma and actinomycetoma cases were compared, no statistically significant difference was found between the other parameters except for the occupational groups (*p* > 0.05). Among the occupational groups, the prevalence among farmers was significantly higher (*p*=0.049).

Abbreviations: DM, diabetes mellitus; HT, hypertension; MRSA, methicillin-resistant *Staphylococcus aureus*; MSSA, methicillin-susceptible *Staphylococcus aureus*.

## Data Availability

The data that support the findings of this study are available from the corresponding author upon reasonable request.
